# Dexamethasone for preventing postoperative nausea and vomiting after mastectomy

**DOI:** 10.1097/MD.0000000000021417

**Published:** 2020-07-24

**Authors:** LeiLai Xu, XiaoHong Xie, XiDong Gu

**Affiliations:** Department of Breast Surgery, First Affiliated Hospital of Zhejiang Chinese Medical University, Hangzhou, China.

**Keywords:** dexamethasone, mastectomy, meta-analysis, postoperative nausea and vomiting, visual analog scale score

## Abstract

**Background:**

Postoperative nausea and vomiting (PONV) is a common complication after mastectomy. Although many researches have been studied the prophylactic effect of antiemetics, none of the results are effective. To overcome this problem, dexamethasone was used to relieve the occurrence of PONV. Since concerns about steroid-related morbidity still remain, We carried out a meta-analysis to evaluate the impact of prophylactic dexamethasone on PONV, post-operative pain undergoing mastectomy.

**Methods:**

Literature search was conducted through PubMed, Web of Science, EMBASE, MEDLINE, and Cochrane library database till June 2019 to identify eligible studies. Meanwhile, we also consulted some Chinese periodicals, such as China Academic Journals, Wanfang and Weipu. The research was reported according to the preferred reporting items for systematic reviews and meta-analysis guidelines. Randomized controlled trials were included in our meta-analysis. Meanwhile, the assessment of the risk of bias was conducted according to the Cochrane Handbook for Systematic Reviews of Interventions version. The pooled data are processed by software RevMan 5.3.

**Results:**

Four studies with 490 patients were enrolled to this meta-analysis. Our study demonstrated that the dexamethasone group was significantly more effective than the placebo group in term of PONV (risk ratio [RR] = 0.46, 95% confidence intervals [CI]: 0.30–0.70, *P* = .0003), nausea (RR = 0.26, 95% CI: 0.10–0.68, *P* = .006) and vomiting (RR = 0.15, 95% CI: 0.04∼0.55, *P* = .004). The visual analog scale score was significantly diminished at 1 hour (weighted mean difference = -1.40, 95% CI: -1.53 to -1.26, *P* < .00001) in the dexamethasone group, while, no statistically significant difference was observed between the two groups in terms of visual analog scale at 24 hours (weighted mean difference = -0.56, 95% CI: -1.24 to 0.13, *P* = 0.11).

**Conclusion:**

Not only does Dexamethasone reduce the incidence of PONV but also decreases postoperative pain. However, we still need larger samples and higher quality studies to determine the relationship between symptoms and administration time to reach the conclusion.

**Trial registration number:**

PROSPERO CRD 42018118575

## 1 Introduction

Postoperative nausea and vomiting (PONV) is common side effect, especially for women who once underwent mastectomy are at high risk of PONV. Researches showed that 60% to 80% of the reported incidents came from patients not receiving antiemetic medication.^[1–3]^ Emetic episodes predispose patients to numerous complications, such as gastric aspiration, wound dehiscence, psychological distress, delayed recovery and discharge times.^[4]^ That's why women who schedule for breast surgery intend to use prophylactic antiemetics to prevent PONV ahead of time.

Several previous studies have argued that the use of dexamethasone is one potent prophylaxis for PONV.^[1–4]^ The mechanism is possibly involved in endogenous prostaglandin and opioid production. Preoperative small dose of dexamethasone can not only relieve the occurrence of PONV, but also effectively reduce postoperative pain.^[5,6]^ Because of its potential side effects, the debate of the application of preoperative dexamethasone never ends.^[7]^ Wattwil et al^[8]^ reported that 4 mg dexamethasone was effective in preventing PONV after breast surgery but found no effect on postoperative pain. Gómez-Hernández et al^[9]^ found a significant reduction in postoperative complications including nausea, vomiting and pain after the preoperative application of 8 mg dexamethasone for patients undergoing surgery for breast cancer. However, some studies have reported the potential side effects of dexamethasone, such as injured sleep quality, increased risk of infection and early postoperative blood glucose elevation.^[10]^ Until now, whether dexamethasone can relieve PONV effectively or reduce pain still remain controversial. Therefore, we carried out this meta-analysis to determine the effect of prophylactic dexamethasone on PONV and pain after mastectomy by conducting a meta-analysis of the available evidence.

## 2 Methods

Our research was reported according to the preferred reporting items for systematic reviews and meta-analysis guidelines.^[11,12]^ The study was registered in PROSPERO, registration number CRD 42018118575.

### 2.1 Search strategy and study selection

Literature search was conducted through PubMed, Web of Science, EMBASE, MEDLINE, and Cochrane library database till June 2019 to identify eligible studies. meanwhile, we also consulted some Chinese periodicals, such as China Academic Journals, Wanfang and Weipu. The following terms were used for Medical Subject Headings and free-text searching: mastectomy, dexamethasone or methylfluorprednisolone or hexadecadrol, PONV, nausea, vomiting, postoperative. The “related articles” facility in PubMed was used to broaden the search, and all abstracts, studies, and citations retrieved were reviewed. In addition, we attempted to identify other studies by searching the reference sections of relevant papers and by contacting known experts in the field. We did not impose any language restriction or seek unpublished data or trials.

### 2.2 Inclusion and exclusion criteria

To be included in the analysis, studies had to be randomized controlled trials (RCTs), evaluating the prophylactic effect of dexamethasone compared with placebo without other anti-emetics on PONV in patients undergoing mastectomy. In addition, there had to be clear reporting of the patient inclusion and exclusion criteria, anesthetic technique, protocol for administration of the experimental drugs, and a definition and evaluation of nausea and vomiting. Reports were excluded from the analysis if patients were undergoing other surgical procedures concomitantly, dexamethasone was administered via the oral or rectal and not the intravenous route, anesthesia techniques were different, the outcomes of interest were not clearly reported, it was impossible to extract or calculate the appropriate data from the published results, or there was duplicate reporting of patient cohorts.

### 2.3 Literature selection

All searched studies were imported into endnote X9 and duplicate documents were deleted. And then, according to the title and abstract of the literature, irrelevant literatures were excluded. At last, 2 researchers removed the literature that meets the exclusion criteria. When the opinion is inconsistent, we discussed the decision with the senior reviewer.

### 2.4 Date extraction

Researchers independently distill the relevant items. The data include first author names, published year, sample size, anesthetic technique, interventions. The primary outcome was the occurrence of nausea, vomiting and PONV during the immediate 24 hour postoperative period. The secondary outcomes were pain score.

### 2.5 Assessment of study quality

Two investigators independently evaluated the literature-related risk of bias according to the Cochrane Handbook for Systematic Reviews of Interventions version. The evaluation criteria include the following 7: sequence generation, allocation concealment, blinding of participants, blinding of outcome assessor, incomplete outcome data, reporting bias, and other bias. If there is a discrepancy between the evaluations, a 3rd reviewer should be asked to join the discussion.

### 2.6 Data analysis

Analysis was carried out using the Review Manager, version 5. When necessary, standard deviations were estimated from the provided confidence interval limits, standard error, or range values. Scales were brought to the same units before pooling the data. Effect sizes of dichotomous outcomes were reported a risk ratios (RR) by using the Mantel-Haenszel method. Continous outcomes were reported as the mean difference by using inverse variance method. The precision of the effect sizes were reported as 95% confidence intervals (CIs). The data were pooled only where there was adequate clinical and methodologic similarity among studies. Statistical heterogeneity was assessed by the *I*^2^ test with *I*^2^ quantifying the proportion of the total outcome variability attributable to variability among studies. Subgroup analysis was done to address heterogeneity. The potential of publication bias was evaluated using Begg and Egger tests and visual inspection of the funnel plot.^[13,14]^

## 3 Results

### 3.1 Search result

**Figure 1:**
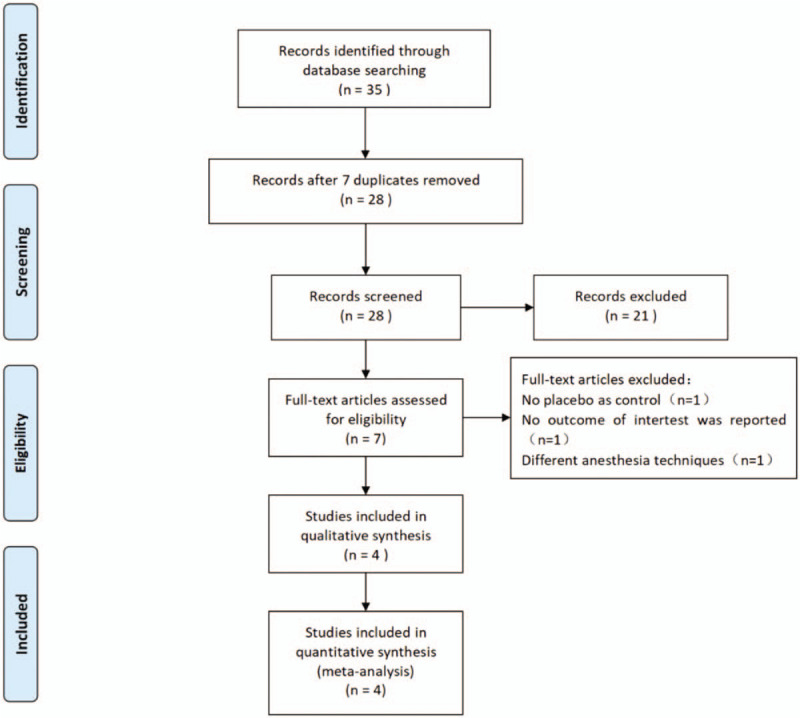
Search results and the selection procedure.

A flowchart describing the process of screening and selection of trials was shown in Figure 1. Our initial search yielded 35 studies, a total of 28 studies were remained after removal of duplicates. According to the titles and abstracts, 21 studies were excluded. The last 7 studies were evaluated by reading the full texts, 1 study was removed for failing to satisfy the related information, 1 administered the non-placebo experimental drugs, and one was different anesthesia techniques. Finally, 4 RCTs^[9,15–17]^ were selected in meta-analysis.

### 3.2 Description of included studies

Some of the basic information of the studies was showed in Table 1. A total of 490 samples were included, 245 patients were in the dexamethasone group, and 245 were in the control group. Intravenous dexamethasone was received in experimental group,while placebo was received in control group. Most of the trials evaluated patients in American Society of Anesthesiologists physical status classes I (normal) and II (mild systemic disease, not activity-limiting).

**Table 1:**
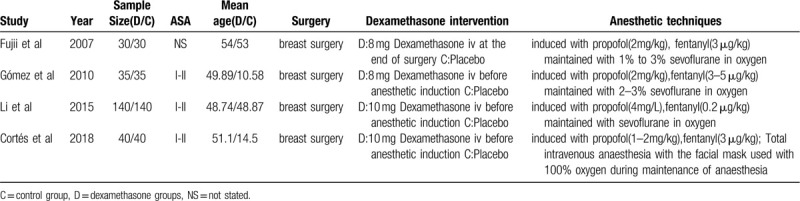
Characteristics of the individual studies included in this meta-analysis.

Anesthesia was induced with propofol and fentanyl intravenously in all studies.^[9,15–17]^ a single dose of dexamethasone (8 or 10 mg) was administered intravenously before anesthetic induction in four studies.^[9,16,17]^ Fujii^[15]^ administered it at the end of surgery. Three studies reported PONV,^[9,15,16]^ two studies reported only the combined incidence of nausea and vomiting,^[15,17]^ two studies reported the pain score.^[9,17]^

### 3.3 Risk of bias assessment

**Figure 2:**
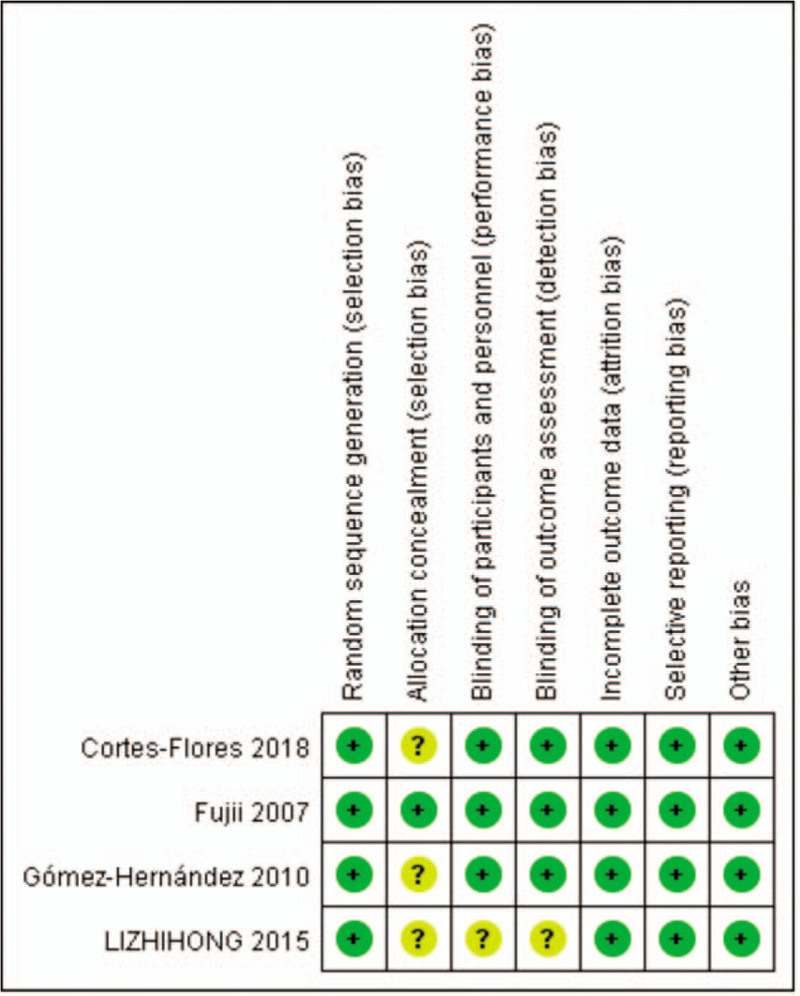
Methodological quality of the randomized controlled trials.

**Figure 3:**
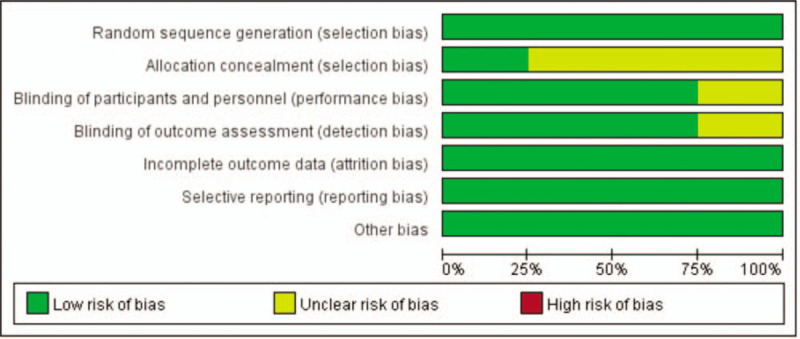
Risk of bias.

The risk of bias summary and risk of bias graph are provided in Fig. 2. Four of the studies reported genuine methods of randomization.^[9,15–17]^ Only 1 study clearly described the method of allocation concealment.^[15]^ Three studies provided blinding of participants and personnel.^[9,15,17]^ Blinding of outcome assessment was unclear in 1 studies^[16]^ and low risk in three studies,^[9,15,17]^ the rest all had low risk of bias. In all studies, the risk of each biased project is a percentage. Percentage represents the degree of risk of bias for each item to varying degrees (Fig. 3).

### 3.4 Primary outcomes

#### 3.4.1 Dexamethasone versus placebo: incidence of PONV

**Figure 4:**
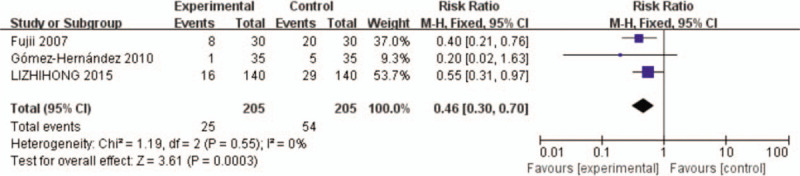
The incidence of PONV after mastectomy. PONV = postoperative nausea and vomiting.

Three studies^[9,15,16]^ recorded the incidence of PONV. There was no significant heterogeneity between Three studies (*x*^2^ = 1.19, df = 2, *I*^2^ = 0%, *P* = .55). Therefore, the fixed-effects model was applied to count data. The results of the analysis show that the incidence of PONV in control group was significant higher than in dexamethasone group (RR = 0.46, 95% CI: 0.30∼0.70, *P* = .0003, Fig. 4).

#### 3.4.2 Dexamethasone versus placebo: incidence of nausea

**Figure 5:**
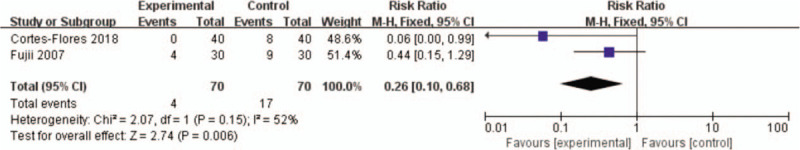
The incidence of nausea after mastectomy.

The incidence of nausea after surgery was provided by two studies.^[15,17]^ There was no significant heterogeneity between 2 studies (*x*^2^ = 2.07, df = 1, *I*^2^ = 52%, *P* = .15). The results of the analysis show that the incidence of nausea in control group was significant higher than in dexamethasone group (RR = 0.26, 95% CI: 0.10∼0.68, *P* = .006, Fig. 5).

#### 3.4.3 Dexamethasone versus placebo: incidence of vomiting

**Figure 6:**
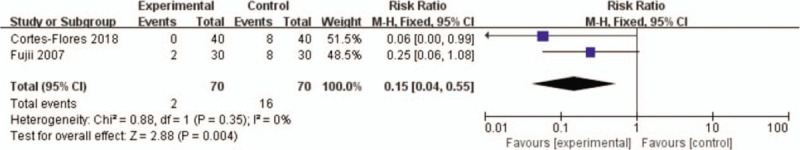
The incidence of vomiting after mastectomy.

The incidence of vomiting was reported in two studies.^[15,17]^ There was no significant heterogeneity between 2 studies (*x*^2^ = 0.88, df = 1, *I*^2^ = 0%, *P* = 0.35). The results of the analysis show that the incidence of vomiting in control group was significant higher than in dexamethasone group (RR = 0.15, 95% CI: 0.04∼0.55, *P* = .004, Fig. 6).

#### 3.4.4 Dexamethasone versus placebo: visual analog scale (VAS) score at one hour

**Figure 7:**
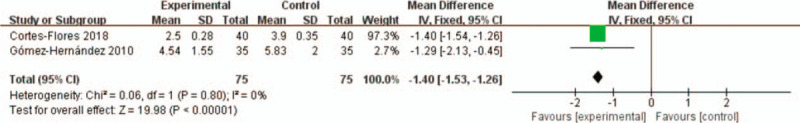
VAS score at 1 h after mastectomy. VAS = visual analog scale.

Two studies^[9,17]^ assessed 1 hour VAS scores after breast surgery. Because of no obvious heterogeneity between the studies (*x*^2^ = 0.06, df = 1, *I*^2^ = 0%, *P* = .80), the fixed-effects model was selected for using. Data summary analysis shows that VAS scores at 1 hour in experimental group were significantly lower than which in control group (weighted mean difference  = -1.40, 95% CI: -1.53∼-1.26, *P* < .00001, Fig. 7).

#### 3.4.5 Dexamethasone versus placebo: VAS score at 24 hours

**Figure 8:**
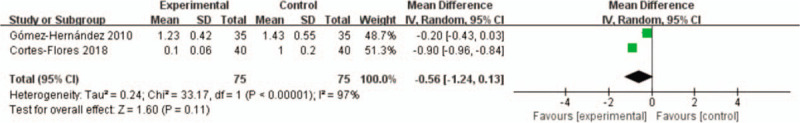
VAS score at 24 h after mastectomy.w. VAS = visual analog scale.

The outcome of VAS scores at 24 hours after surgery was provided by all 2 studies.^[9,17]^ No heterogeneity significantly exists between studies, so a fixed-effects model was applied (*x*^2^ = 33.17, df = 1, *I*^2^ = 97%, *P* < .00001). The results of the analysis show that VAS scores at 24 hours in control group were higher than in dexamethasone group (weighted mean difference = -0.56, 95% CI: -1.24∼0.13, *P* = 0.11, Fig. 8).

## 4 Discussion

Preventing PONV started to become important since Apfel et al realized that the fear of PONV is much more than postoperative pain by conducting preoperative interviews with patients.^[18]^ A recent systematic review demonstrated that prophylactic dexamethasone decreased the incidence of PONV after total hip arthroplasty relative to placebo.^[19]^ Unlike other surgical interventions, mastectomy performed under general anesthesia is associated with a markedly high incidence of PONV.

Our results for the first time compared the effectiveness of dexamethasone with that of placebo in the prevention of PONV after mastectomy. This review presents evidence that the preoperative administration of dexamethasone for patients undergoing mastectomy offers a significant benefit over placebo in reducing the incidence of PONV, nausea, vomiting.

Our study also showed that the incidence of PONV in control group was significant higher than in dexamethasone group. The pooled RR of PONV was 0.46, which is a 54% reduction. The pooled risk difference of 14% translates into a number-needed to treat of about 7, that is, on average there would have been one fewer case of PONV for every 7 persons treated with dexamethasone compared to placebo.

The current study showed that dexamethasone could effectively reduce neither postoperative nausea nor vomiting. Kleif et al^[20]^ found that dexamethasone did not effectively relieve the nausea and vomiting after laparoscopy for suspected appendicitis. However, dexamethasone as a long-acting glucocorticoid has been widely used in PONV. The addition of a single dose of 8 mg intravenous dexamethasone at the induction of anaesthesia was reported to reduce both the incidence of PONV at 24 hours,^[21]^ which is consistent with our statistical results. In our meta-analysis, dexamethasone can significantly reduce the PONV after mastectomy. Kehlet et al^[22]^ demonstrated that dexamethasone can reduce the incidence of PONV by central antiemetic effect.

Pain after mastectomy is another major cause of discomfort. VAS score is the primary assessment of our meta-analysis. Christensen et al^[23]^ showed that dexamethasone did not effectively relieve the pain after total knee arthroplasty. However, Xu H et al^[24]^ showed that intravenous injection of dexamethasone can significantly reduce postoperative pain, which is aligned with our statistical results. In our meta-analysis, dexamethasone can significantly reduce the postoperative pain score at the first hours after mastectomy, but the VAS score is not significantly diminished at 24 hours.

Dexamethasone works better when given before surgery. The result is likely to be related to moderating surgery-related inflammation.^[25]^ Three studies^[9,16,17]^ in this review administered dexamethasone before induction, and another study^[15]^ administered it at the end of surgery. There was no evidence that the treatment effect was postponed by the late administration, but this needs to be further studied.

Admittedly, our meta-analysis still has limitations as follows. First, four RCTs were included in the meta-analysis, the amount of sample data is relatively small. Thus, more RCTs in the later stages will be needed. Second, Only 3 studies reported PONV, and only two studies reported nausea or vomiting, VAS score at one or 24 hours, so publication bias cannot be assessed. In addition, we were having troubles in determining the efficacy of dexamethasone compared with other commonly used antiemetics such as ondansetron or serotonin 5HT3 receptor antagonist. Pregnant women, diabetics, and obese patients were excluded in three studies.^[9,17]^ Therefore, the effect and the safety of administration of dexamethasone in these patients needs further evaluation.

## 5 Conclusions

Dexamethasone not only reducing the incidence of PONV, nausea and vomiting, but also reduces postoperative pain scores. However, we still need large sample size and high quality studies to explore the relationship between symptoms and administration time to give the final conclusion.

## Author contributions

**Data curation:** Leilai Xu

**Formal analysis:** Leilai Xu

**Methodology:** Leilai Xu

**Resources:** XiDong Gu, XiaoHong Xie

**Software:** Leilai Xu

**Supervision:** XiDong Gu, XiaoHong Xie

**Writing – original draft:** Leilai Xu

**Writing – review & editing:** Leilai Xu, XiDong Gu
